# Reliability of routine clinical measurements of neonatal circumferences and research measurements of neonatal skinfold thicknesses: findings from the Born in Bradford study

**DOI:** 10.1111/j.1365-3016.2010.01181.x

**Published:** 2011-01-24

**Authors:** Jane West, Ben Manchester, John Wright, Debbie A Lawlor, Dagmar Waiblinger

**Affiliations:** 1 Bradford Institute for Health Research, Bradford Royal InfirmaryBradford; 2 School of Medicine, University of LeedsLeeds; 3 MRC Centre for Causal Analyses in Translational Epidemiology, Department of Social Medicine, University of BristolBristol, UK

**Keywords:** birth head circumference, mid upper arm circumference, abdominal circumference, skinfold thicknesses, neonatal anthropometry, reliability, Born in Bradford

## Abstract

West J, Manchester B, Wright J, Lawlor DA, Waiblinger D. Reliability of routine clinical measurements of neonatal circumferences and research measurements of neonatal skinfold thicknesses: findings from the Born in Bradford study. *Paediatric and Perinatal Epidemiology* 2011.

Assessing neonatal size reliably is important for research and clinical practice. The aim of this study was to examine the reliability of routine clinical measurements of neonatal circumferences and of skinfold thicknesses assessed for research purposes. All measurements were undertaken on the same population of neonates born in a large maternity unit in Bradford, UK. Technical error of measurement (TEM), relative TEM and the coefficient of reliability are reported.

Intra-observer TEMs for routine circumference measurements were all below 0.4 cm and were generally within ±2-times the mean. Inter-observer TEM ranged from 0.20 to 0.36 cm for head circumference, 0.19 to 0.39 cm for mid upper arm circumference and from 0.39 to 0.77 cm for abdominal circumference. Intra and inter-observer TEM for triceps skinfold thickness ranged from 0.22 to 0.35 mm and 0.15 to 0.54 mm, respectively. Subscapular skinfold thickness TEM values were 0.14 to 0.25 mm for intra-observer measurements and 0.17 to 0.63 mm for inter-observer measurements. Relative TEM values for routine circumferences were all below 4.00% but varied between 2.88% and 14.23% for research skinfold measurements. Reliability was mostly between 80% and 99% for routine circumference measurements and ≥70% for most research skinfold measurements.

Routine clinical measurements of neonatal circumferences are reliably assessed in Bradford. Assessing skinfolds in neonates has variable reliability, but on the whole is good. The greater intra-observer, compared with inter-observer, reliability for both sets of measurements highlights the importance of having a minimal number of assessors whenever possible.

## Introduction

Demands for information regarding size at birth are increasing as we seek to understand more about the determinants, and the short- and long-term effects of variation in birth size. While a large amount of research concerning the effects of neonatal size has used routinely collected data from clinical records, in particular birthweight, there is increasing interest in other measurements of size that might provide more precise estimates of fat vs. lean mass, fat distribution and head circumference (as a possible proxy indicator of neurological development). Head, abdominal and mid upper arm (MUA) circumference are now measured more frequently in the UK and many other countries, but little is known about the reliability of these measures when assessed in routine clinical practice.

Assessments of neonatal body composition using either air displacement plethysmography^1^ or magnetic resonance imaging^2^ are expensive and thus, to date, have only been assessed in research studies of relatively small sample sizes. Skinfold thicknesses are considered to be a more direct way of measuring fat than weight and ratios of central (subscapular) to peripheral (e.g. triceps) skinfolds are used to indicate central distribution of fat. These are not prohibitively expensive and can be assessed in large epidemiological studies.^3^ However, if these measurements are less reliably assessed in neonates than routine measurements the potential benefit of a more direct measure of fat (compared with using for example routinely collected weight or abdominal circumference) might be mitigated by their lack of reliability. To our knowledge, only one previous study has examined the reliability of neonatally assessed skinfold thicknesses^4^ and we are not aware of any previous study that has examined the reliability of routinely measured neonatal circumferences and neonatal skinfolds assessed for research purposes within the same study population.

The aim of this paper is to examine the reliability of routinely collected neonatal circumferences and research collected neonatal skinfold measurements. We have not assessed the reliability of birthweight because this is less prone to human error and relies upon the scales being used in hospitals and how frequently they are calibrated. Furthermore, birthweight is assessed immediately at birth on the labour ward where it would have been impossible for us to introduce reliability assessments for our study without potentially interfering with clinical management.

## Methods

### Population

The Born in Bradford study (BiB) is a prospective multi-ethnic birth cohort study investigating the health and development of babies born in Bradford, UK, throughout their childhood and adolescence. Details of the study methodology have been reported.^5^ In summary, all women booked to give birth at Bradford Teaching Hospitals NHS (National Health Service) Foundation Trust were asked to participate in BiB. Recruitment began in 2007 and will end in 2010. Data collection in the study consists of abstraction of data from routine clinical records, questionnaires administered to parents and additional research measurements and collection of biological specimens from parents and offspring. Neonatal anthropometric measurements for BiB were either abstracted from routinely collected clinical measurements undertaken as part of the first baby examination (head, MUA and abdominal circumference) or were additional measurements conducted only on BiB study participants (subscapular and triceps skinfold thickness measurements). Ethical approval was obtained from the Local Research Ethics Committee. We undertook tests of the reliability of neonatal anthropometric measurements collected at intervals between September 2007 and September 2009.

### Routine clinical measurements of circumferences

In Bradford, head, MUA and abdominal circumference are collected by a paediatrician or specially trained midwife, as part of the routine neonatal examination within the first 24 h following delivery. These measurements are obtained for all babies regardless of whether they are enrolled in the BiB study. Paediatricians and midwives are trained in measurement technique by a consultant paediatrician according to written guidelines as part of their induction to the neonatal unit. Measurements are taken using Lasso-o tapes specially manufactured by Harlow Printing Ltd (South Shields, UK) to accommodate small arm circumferences. A new tape measure is used for each baby in line with hospital policy to minimise infection risk. Data are entered into the hospital's electronic records system (Eclipse).

### Research collected neonatal skinfold measurements

Subscapular and triceps skinfold measurements were collected specifically for the BiB project and only recorded for babies enrolled in the study. Measurements were obtained using Tanner/Whitehouse Calipers (Holtain Ltd) by specially trained BiB study administrators according to a written protocol and always on the left side of the body. Equipment was recalibrated every 12 months. Most skinfold measurements were obtained within the first 24 h following delivery. Rarely some measurements were recorded after this time but were within 72 h of delivery and prior to discharge. Training was delivered at regular intervals and periodic monitoring (monthly) continued throughout the data collection period. Skinfold measurements were entered into the Eclipse electronic records system.

### Reliability assessments

- Routine clinical measurements of neonatal circumferences:
  Both intra- and inter-observer reliability assessments were undertaken using convenience samples. Intra-observer reliability (assessing the extent to which the same clinician when measuring the same infant a second time after a time interval would obtain the same result as their first measurement) was assessed once during the data collection period on a sample of 29 infants. Three paediatricians were accompanied by an independent observer (B. M.) over a 1-week period and measured 23, 4 and 2 infants, respectively, twice. The two measurements were completed with an approximate 5 min time interval by asking the paediatricians to measure each infant, once at the start of the neonatal examination and once at the end. The written record of the first measurement was removed once this was completed so that it was not available for the clinician to see as they performed the second measurement.
  Inter-observer reliability (assessing the extent to which two or more clinicians would get the same result when measuring the same individual) was assessed throughout the data collection period. The measurements taken by the paediatrician or midwife were repeated by a trained assessor (J. W.), who was blind to the initial measurement, within 3 h of the first examination. A total of 24 paediatricians and 8 midwives collected measurements during the data collection period and reliability data were obtained for 6 paediatricians. Replicate recordings were obtained on 10 infants, different to those used for testing intra-observer reliability.
- Research collected neonatal skinfold measurements:
  A total of 10 study administrators collected skinfold measurements during the data collection period and both intra- and inter-observer reliability assessments were undertaken. Intra-observer reliability was assessed using a convenience sample once during the first year of data collection on a sample of 40 infants. Project workers recorded measurements twice usually in the presence of an assessor (J. W.), who removed the initial measurement results once these were completed. On a small number of occasions (20% of the total) it was not possible for the assessor to be present in which case the study administrators recorded their own repeat measurements. Repeat measurements were taken approximately 5 min after the initial recording. The number of infants measured for intra-observer calculations ranged between 4 and 10.
  Inter-observer assessments were performed on a convenience sample of 100 infants (10 for each of the 10 administrators) throughout the 2-year data collection period. Ten infants, different from those included in the intra-observer testing, were measured both by one of the administrators and then repeated by an observer (J.W.) within 5 min of the initial recording. This procedure was repeated for each of the 10 administrators.

### Analysis

#### Justification for the reliability measures used

There are a number of different methods available for measuring reliability. Several of these assess somewhat different aspects of reliability and there is not agreement on which is the best measure for assessing reliability in neonatal anthropometry. We used three measurements that assess different aspects of reliability and compared whether our conclusions would differ depending upon which of these were used. We used the technical error of measurement (TEM), the relative TEM and the coefficient of reliability (R). The TEM measures the standard deviation between repeated measurements^6^ using the same units of measurement. It thus provides a measure of the spread of repeat measurements, the smaller the TEM the more reliable the assessment. The World Health Organization (WHO)^7^ suggest that where an expert assessor is available, acceptable TEM cut-offs should be based on the expert's intra-observer TEM, TEM values for both intra- and inter-observer reliability for others in the study should lie within ±2 times the expert's intra-observer TEM. Where an expert is not available, the average of well-trained observers can be used to set acceptable limits. We took this latter approach here, because for the routine circumference measurements there was no individual paediatrician or midwife who could be considered more experienced than all the others and for the research skinfold measurements the external observer was trained at the same time as the study administrators and so could not be considered to be more expert than them. For the routine circumference measurements each individual paediatrician TEM was compared with the average of all paediatricians assessed during the study period. For the research skinfolds each assessor was compared to the average of all assessors. The relative TEM is a measure of the coefficient of variation and provides an estimate of the size of the error relative to the size of the measurement.^6^ R estimates the proportion of variance not due to error.^7^ While there is no defined threshold for an acceptable level of R,^8^ a cut-off of 90% has been suggested as acceptable for growth measurements.^9^ R of 75% and over has also been suggested as acceptable for skinfold thickness measurements which are typically less reliable than other anthropometric measurements.^7^ Thus, the three measurements used here provide somewhat different assessments of reliability, with the TEM providing an indication of how repeat measurements vary from the mean, the relative TEM providing a measure of size of error (variation from the mean) in relation to the magnitude of the mean and R provides the proportion of variation between measurements that is not due to measurement error.

Finally, we used Bland Altman plots to investigate agreement between the paediatrician or study administrator and the observer. The difference between the measurements was plotted against the mean of the two measurements. We examined these plots for evidence of systematic bias, for example, differences being greater or smaller depending on the overall mean of the two measurements.

Analyses were performed using SPSS version 14.0 for Windows (SPSS Inc., Chicago, IL, USA) and STATA 10 (StataCorp, College Station, TX, USA).

## Results

Results of intra- and inter-reliability tests of routinely collected circumferences are presented in Tables 1 and 2. The intra- and inter-observer TEM values were all within ±2-times the average for all assessors. Relative TEM values were all below 3.5% and R was between 80% and 99% for the majority of routinely collected circumference measurements, though was low (64 and 65%) for two.

**Table 1:** Intra-observer reliability of routine clinical measurements (circumferences)

Measurer	TEM (cm)	Relative TEM (%)	Reliability (%)	Average TEM (cm)
Head circumference				
Clinician 1^a^	0.10	0.28	99	0.12
Clinician 2^b^	0.20	0.57	99	0.12
Clinician 3^c^	0.06	0.17	97	0.12
MUA circumference				
Clinician 1^a^	0.33	2.98	65	0.16
Clinician 2^b^	0.09	0.81	99	0.16
Clinician 3^c^	0.06	0.54	97	0.16
Abdominal circumference				
Clinician 1^a^	0.13	0.40	99	0.11
Clinician 2^b^	0.17	0.56	99	0.11
Clinician 3^c^	0.03	0.10	97	0.11

Results based on ^a^23,^b^4 and^c^2 replicate measurements.

Average TEM is the average of all the measurers assessed during the study period.

MUA, mid upper arm, TEM, technical error of measurement.

**Table 2:** Inter-observer reliability of routine clinical measurements (circumferences)^a^

Measurer/ anthropometry	TEM (cm)	Relative TEM (%)	Reliability (%)	Average TEM (cm)
Head circumference				
Clinician 1	0.29	0.86	94	0.28
Clinician 2	0.35	1.01	96	0.28
Clinician 3	0.36	1.04	92	0.28
Clinician 4	0.20	0.60	95	0.28
Clinician 5	0.25	0.74	95	0.28
Clinician 6	0.21	0.61	95	0.28
MUA circumference				
Clinician 1	0.29	2.57	78	0.29
Clinician 2	0.39	3.39	84	0.29
Clinician 3	0.22	1.98	95	0.29
Clinician 4	0.19	1.77	95	0.29
Clinician 5	0.35	3.40	64	0.29
Clinician 6	0.28	2.61	87	0.29
Abdominal circumference				
Clinician 1	0.54	1.71	87	0.69
Clinician 2	0.58	1.75	93	0.69
Clinician 3	0.39	1.22	96	0.69
Clinician 4	0.74	2.42	81	0.69
Clinician 5	0.63	2.11	86	0.69
Clinician 6	0.77	2.48	88	0.69

a Results based on 10 replicate measurements for each clinician.

Average TEM is the average of all the measurers assessed during the study period.

MUA, mid upper arm, TEM, technical error of measurement.

Intra- and inter-observer reliability of research skinfold measurements are shown in Tables 3 and 4. Intra- and inter-observer TEM were within ±2-times the average for both skinfolds. The relative TEM for the intra-observer comparisons for the skinfolds were <5% for the majority and <7% for all. The inter-observer relative TEM, however, varied markedly from 3.27% to 14.23%. R was ≥70% for all but one of the study administrators for whom it was 64%.

**Table 3:** Intra-observer reliability of research collected measurements (skinfolds)

Measurer	TEM (mm)	Relative TEM (%)	Reliability (%)	Average TEM (mm)
Subscapular skinfold				
Administrator 1^a^	0.16	3.00	76	0.20
Administrator 2^b^	0.19	3.81	97	0.20
Administrator 3^c^	0.25	5.98	78	0.20
Administrator 4^b^	0.14	2.88	98	0.20
Administrator 5^b^	0.25	5.09	94	0.20
Triceps skinfold				
Administrator 1^a^	0.26	4.25	94	0.26
Administrator 2^b^	0.22	4.06	97	0.26
Administrator 3^c^	0.26	5.29	64	0.26
Administrator 4^b^	0.35	6.98	87	0.26
Administrator 5^b^	0.22	4.16	94	0.26

Results based on ^a^6,^b^10 and^c^4 replicate measurements.

Average TEM is the average of all the measurers assessed during the study period.

TEM, technical error of measurement.

**Table 4:** Inter-observer reliability of research collected measurements (skinfolds)^a^

Measurer	TEM (mm)	Relative TEM (%)	Reliability (%)	Average TEM (mm)
Subscapular skinfold				
Administrator 1	0.38	7.07	77	0.42
Administrator 2	0.17	3.55	97	0.42
Administrator 3	0.49	9.68	79	0.42
Administrator 4	0.63	14.23	75	0.42
Administrator 5	0.42	9.01	71	0.42
Administrator 6	0.22	4.15	94	0.42
Administrator 7	0.55	11.99	53	0.42
Administrator 8	0.31	6.68	86	0.42
Administrator 9	0.61	10.64	71	0.42
Triceps skinfold				
Administrator 1	0.26	4.47	88	0.35
Administrator 2	0.15	3.27	97	0.35
Administrator 3	0.47	8.75	80	0.35
Administrator 4	0.37	7.85	89	0.35
Administrator 5	0.51	10.32	68	0.35
Administrator 6	0.25	3.93	96	0.35
Administrator 7	0.54	10.39	62	0.35
Administrator 8	0.24	4.79	83	0.35
Administrator 9	0.38	6.55	89	0.35

a Results based on 10 replicate measurements per administrator.

Average TEM is the average of all the measurers assessed during the study period.

TEM, technical error of measurement.

Tables 1–4 demonstrate two additional points. First, they illustrate that the lowest levels of relative TEM do not correspond to the highest levels of R and therefore show that these two measurements are picking up somewhat different aspects of reliability. Second, for both routine clinical measurements of circumferences and research measurements of skinfolds intra-observer reliability is better than inter-observer reliability.

Bland Altman plots (Figures S1–S36, Supporting information) show that all mean differences were close to zero. Data points fell close to the line of mean difference and were spread evenly across either side of the line suggesting no systematic bias although they should be viewed with the understanding that each plot was based on just 10 data points.

## Discussion

Our results show that routinely assessed clinical measurements of head, MUA and abdominal circumference are measured reliably in this area. Skinfold thicknesses measured specifically for our research study were less reliably measured, which has implications for their use in ours and other research studies. For both sets of measurements intra-observer reliability was better than inter-observer reliability, highlighting the importance of minimising the number of individuals completing measurements in any cohort.

Reliability assessment of neonatal measurements is not straightforward and very few studies have attempted this. First, there is no universal consensus regarding the most appropriate statistical method.^8,10–12^ In our study, we used three commonly used measurements that assess different aspects of reliability. TEM tended to universally suggest all measurements were reliable whereas both relative TEM and R suggested some differences. We would therefore suggest that TEM may not be the best measurement for assessing reliability of neonatal anthropometry as it may reassure when other measurements indicate a need for concern and further training. Relative TEM and R were not completely consistent (i.e. they did not rank observers in exactly the same order) and therefore we would recommend that both are used in future studies or audits in order to identify observers or measurements where further training is needed.

Second, there are widespread inconsistencies in the interpretation of results, with results often interpreted by comparison to previously published studies that have used different protocols, observers, subjects and equipment.^7^ Papers frequently report that reliability is within acceptable ranges, but because this is often done on the basis of comparison to previous studies and acceptable ranges should take account of the differing characteristics of the measurements and population, such conclusions should be treated with caution.^8^ In our analyses we have followed WHO guidance and compared each individual with the average of all of them, rather than compare with any published study. Third, the robustness of assessments of reliability of neonatal measurements is likely to be affected by small study sample numbers. Ideally, one would like to repeatedly assess the reliability on large numbers throughout the whole of the study period in all birth cohort studies. In practice, this is extremely difficult because of the short time period between birth and discharge and the need to prioritise clinical care over any research needs.

We believe that our study is unique in assessing the reliability of neonatal routinely collected circumferences and measurements of skinfolds specifically collected for research within the same study population. The greater reliability of the routinely collected circumferences compared with the skinfold thicknesses collected specifically for the research study are likely to reflect the fact that skinfolds are known to be more difficult and less likely to be reliably assessed than other anthropometric measurements.^7^

Based on R, reliability for one administrator (Administrator 7) fell short of acceptable limits but interestingly this individual had been identified for retraining through ongoing monitoring prior to these results. This highlights the importance of both detailed protocols and repeated training, as well as monitoring of reliability, in research studies. These results will be helpful in future analyses using these data where we will be able to control for the individual assessors to improve precision and also undertake sensitivity analyses with reasonable assessments of measurement error and variation between assessors.

In a separate study of BiB participants it has been demonstrated that measurements of weight, height and head circumference routinely collected by health visitors in later infancy can be accurately and reliably measured after training.^13^ A different British cohort found that routine measurements of weight and height in infancy (from age 8 months) were accurate when compared with measurements on the same individuals conducted in research clinics after training of the health visitors collecting the routine data.^14^ These findings, together with ours presented here, have important implications for clinical practice and epidemiological research. Use of routinely collected clinical data in research would avoid the costly duplication of data collection by researchers, provide confidence in large population datasets and help bring together the worlds of research and practice.

### Strengths and limitations

We aimed to undertake replicate measurements on a minimum of 10 infants for each measurer. This was difficult to achieve for some assessments due to clinical pressures and the importance of always allowing clinical practice to take precedence over our research. Three paediatricians participated in the intra-observer assessment on 23, 4 and 2 infants, respectively. Two of the paediatricians were called to clinical incidents during these assessments resulting in the small number of infants measured. All study administrators taking measurements at the time of the intra-observer assessments were included in the assessment. Again, we aimed to obtain replicate measurements on ten infants but this was only possible for three administrators (because of periods of leave), four and six infants were measured for the remaining two administrators. Adequate numbers (10 infants) were obtained for all other assessments.

### Conclusions

Our findings demonstrate that routinely collected neonatal measurements of circumferences are reliable in Bradford. Neonatal skinfold thicknesses for research are less reliable than routine circumference measurements but on the whole had reasonable reliability. Whenever possible minimising the number of staff used to assess neonatal anthropometry will improve reliability and it is always going to be important to continually train and retrain assessors, as well as monitoring reliability.

## Notes

**Figures S1–S9** Subscapular skinfoldthickness (mm) for Administrators 1–9 respectively **Figures S10–S19** Triceps skinfoldthickness (mm) for Administrators 1–9 respectively **Figures S20–S24** Head circumference (cm)for Clinicians 1–6 respectively **Figures S25–S30** Arm circumference(cm) forClinicians 1–6 respectively **Figures S31–S36** Abdominal circumference (cm) for Clinicians 1-6 respectively
