# Supplementary material for: Reliability of routine clinical measurements of neonatal circumferences and research measurements of neonatal skinfold thicknesses: findings from the Born in Bradford study
Source: Paediatr Perinat Epidemiol. 2011 Jan 24;25(2):164–71. doi: 10.1111/j.1365-3016.2010.01181.x (PMC3532621; doi:10.1111/j.1365-3016.2010.01181.x)
Supplement: Supplementary file 1 — Figures S1–S9 Subscapular skinfoldthickness (mm) for Administrators 1–9 respectively Figures S10–S19 Triceps skinfoldthickness (mm) for Administrators 1–9 respectively Figures S20–S24 Head circumference (cm)for Clinicians 1–6 respectively Figures S25–S30 Arm circumference(cm) forClinicians 1–6 respectively Figures S31–S36 Abdominal circumference (cm) for Clinicians 1-6 respectively [file ppe0025-0164-SD1.doc]

**Supplementary tables**

**Figure S1 Bland-Altman plot for subscapular skinfold thickness (mm) Administrator 1**

**Figure S2 Bland-Altman plot for subscapular skinfold thickness (mm)**

**Administrator 2**

**Figure S3 Bland-Altman plot for subscapular skinfold thickness (mm)**

**Administrator 3**

**Figure S4 Bland-Altman plot for subscapular skinfold thickness (mm)**

**Administrator 4**

**Figure S5 Bland-Altman plot for subscapular skinfold thickness (mm)**

**Administrator 5**

**Figure S6 Bland-Altman plot for subscapular skinfold thickness (mm)**

**Administrator 6**

**Figure S7 Bland-Altman plot for subscapular skinfold thickness (mm)**

**Administrator 7**

**Figure S8 Bland-Altman plot for subscapular skinfold thickness (mm)**

**Administrator 8**

**Figure S9 Bland-Altman plot for subscapular skinfold thickness (mm)**

**Administrator 9**

**Figure S10 Bland-Altman plot for triceps skinfold thickness (mm)**

**Administrator 1**

**Figure S11 Bland-Altman plot for triceps skinfold thickness (mm)**

**Administrator 2**

**Figure S12 Bland-Altman plot for triceps skinfold thickness (mm)**

**Administrator 3**

**Figure S13 Bland-Altman plot for triceps skinfold thickness (mm)**

**Administrator 4**

**Figure S14 Bland-Altman plot for triceps skinfold thickness (mm)**

**Administrator 5**

**Figure S15 Bland-Altman plot for triceps skinfold thickness (mm)**

**Administrator 6**

**Figure S16 Bland-Altman plot for triceps skinfold thickness (mm)**

**Administrator 7**

**Figure S17 Bland-Altman plot for triceps skinfold thickness (mm)**

**Administrator 8**

**Figure S18 Bland-Altman plot for triceps skinfold thickness (mm)**

**Administrator 9**

**Figure S19 Bland-Altman plot for head circumference (cm)**

**Clinician 1**

**Figure S20 Bland-Altman plot for head circumference (cm)**

**Clinician 2**

**Figure S21 Bland-Altman plot for head circumference (cm)**

**Clinician 3**

**Figure S22 Bland-Altman plot for head circumference (cm)**

**Clinician 4**

**Figure S23 Bland-Altman plot for head circumference (cm)**

**Clinician 5**

**Figure S24 Bland-Altman plot for head circumference (cm)**

**Clinician 6**

**Figure S25 Bland-Altman plot for arm circumference (cm)**

**Clinician 1**

**Figure S26 Bland-Altman plot for arm circumference (cm)**

**Clinician 2**

**Figure S27 Bland-Altman plot for arm circumference (cm)**

**Clinician 3**

**Figure S28 Bland-Altman plot for arm circumference (cm)**

**Clinician 4**

**Figure S29 Bland-Altman plot for arm circumference (cm)**

**Clinician 5**

**Figure S30 Bland-Altman plot for arm circumference (cm)**

**Clinician 6**

**Figure S31 Bland-Altman plot for abdominal circumference (cm)**

**Clinician 1**

**Figure S32 Bland-Altman plot for abdominal circumference (cm)**

**Clinician 2**

**Figure S33 Bland-Altman plot for abdominal circumference (cm)**

**Clinician 3**

**Figure S34 Bland-Altman plot for abdominal circumference (cm)**

**Clinician 4**

**Figure S35 Bland-Altman plot for abdominal circumference (cm)**

**Clinician 5**

**Figure S36 Bland-Altman plot for abdominal circumference (cm)**

**Clinician 6**
